# Bat Flies of the Family *Streblidae* (Diptera: Hippoboscoidea) Host Relatives of Medically and Agriculturally Important “Bat-Associated” Viruses

**DOI:** 10.3390/v13050860

**Published:** 2021-05-08

**Authors:** María M. Ramírez-Martínez, Andrew J. Bennett, Christopher D. Dunn, Thomas M. Yuill, Tony L. Goldberg

**Affiliations:** 1 Departamento de Ciencias de la Salud y Ecología Humana, Universidad de Guadalajara, Guadalajara, Autlán CP 48900, Mexico; mmagdalena.ramirez@academicos.udg.mx; 2 Department of Pathobiological Sciences, School of Veterinary Medicine, University of Wisconsin–Madison, Madison, WI 53706, USA; andrew.j.bennett@gmail.com (A.J.B.); cddunn2@wisc.edu (C.D.D.); thomas.yuill@wisc.edu (T.M.Y.); 3 Genomics and Bioinformatics Department, Biological Defense Research Directorate, Naval Medical Research Center–Frederick, Fort Detrick, Frederick, MD 21702, USA

**Keywords:** chiroptera, bat fly, hippoboscoidea, streblidae, nycteribiidae, rhabdoviridae, vesiculovirus, reoviridae, orbivirus, peribunyaviridae, orthobunyavirus

## Abstract

Bat flies (Hippoboscoidea: *Nycteribiidae* and *Streblidae*) are obligate hematophagous ectoparasites of bats. We collected streblid bat flies from the New World (México) and the Old World (Uganda), and used metagenomics to identify their viruses. In México, we found méjal virus (*Rhabdoviridae*; *Vesiculovirus*), Amate virus (*Reoviridae*: *Orbivirus*), and two unclassified viruses of invertebrates. Méjal virus is related to emerging zoonotic encephalitis viruses and to the agriculturally important vesicular stomatitis viruses (VSV). Amate virus and its sister taxon from a bat are most closely related to mosquito- and tick-borne orbiviruses, suggesting a previously unrecognized orbivirus transmission cycle involving bats and bat flies. In Uganda, we found mamucuso virus (*Peribunyaviridae*: *Orthobunyavirus*) and two unclassified viruses (a rhabdovirus and an invertebrate virus). Mamucuso virus is related to encephalitic viruses of mammals and to viruses from nycteribiid bat flies and louse flies, suggesting a previously unrecognized orthobunyavirus transmission cycle involving hippoboscoid insects. Bat fly virus transmission may be neither strictly vector-borne nor strictly vertical, with opportunistic feeding by bat flies occasionally leading to zoonotic transmission. Many “bat-associated” viruses, which are ecologically and epidemiologically associated with bats but rarely or never found in bats themselves, may actually be viruses of bat flies or other bat ectoparasites.

## 1. Introduction

Bat flies (Hippoboscoidea: *Nycteribiidae* and *Streblidae*) are obligate hematophagous ectoparasites that are highly adapted to life on bats [1]. The *Nycteribiidae* occur mainly in the Old World, have lost their eyes and wings over evolutionary time, and have a small thorax relative to their abdomen (due to the loss of flight muscles), giving them a “spider-like” appearance [1,2,3]. In contrast, the *Streblidae* have a worldwide distribution (but with most species occurring in the tropics) and have greater morphological variation than the *Nycteribiidae* [1,4]. The wings of streblid bat flies can be normal (relative to other dipterans), reduced, or absent, the eyes can be small or absent, and the general body form can be flattened laterally or dorsoventrally [4]. The *Nycteribiidae* and *Streblidae* are thought to have originated during a major period of bat diversification between 50 and 30 million years ago [5].

Nycteribiid bat flies host apicomplexan parasites of the genus *Polychromophilus*, the cause of “bat malaria” [6,7], and bacteria of the genus *Bartonella*, some of which are zoonotic [8,9]. Wolkberg and Kaeng Khoi viruses (*Perbunyaviridae*: *Orthobunyavirus*) were isolated from the nycteribiid bat flies *Eucampsipoda africana* Theodor, 1955 and *E. sundaica* Theodor, 1955 in South Africa and China, respectively [10,11,12], and Mahlapitsi virus (*Reoviridae*: *Orthoreovirus*) was isolated from *E. africana* in South Africa [13]. Members of our group have reported a surprising diversity of ledanteviruses (*Rhabdoviridae*: *Ledantevirus*) related to zoonotic agents in nycteribiid bat flies from Uganda, with flies from individual bats hosting multiple divergent viral variants [14]. Fewer reports exist about microbes in streblid bat flies. *Bartonella* sp. bacteria have been detected in streblid bat flies in México [15] and globally [8], and dengue virus (*Flaviviridae*: *Flavivirus*) has been detected in streblid bat flies on vampire bats (*Desmodus rotundus* Geoffroy, 1810) in México [16].

In our previous studies of nycteribiid bat flies [14,17], we hypothesized that highly co-evolved bat ectoparasites might host “bat-associated” viruses, defined as viruses that are ecologically and epidemiologically linked to bats but have rarely or never been found infecting bats. Such “bat-associated” viruses may actually be hosted not by bats themselves, but by other organisms that associate closely with bats in nature, such as bat ectoparasites. Here, we explore this idea further by examining streblid bat flies from the New World (México) and the Old World (Uganda). Our data expand the known diversity of viruses infecting bat flies and show that streblid bat flies do, indeed, host relatives of “bat-associated” viruses important to human and animal health.

## 2. Materials and Methods

### 2.1. Study Sites and Sampling

**Figure 1:**
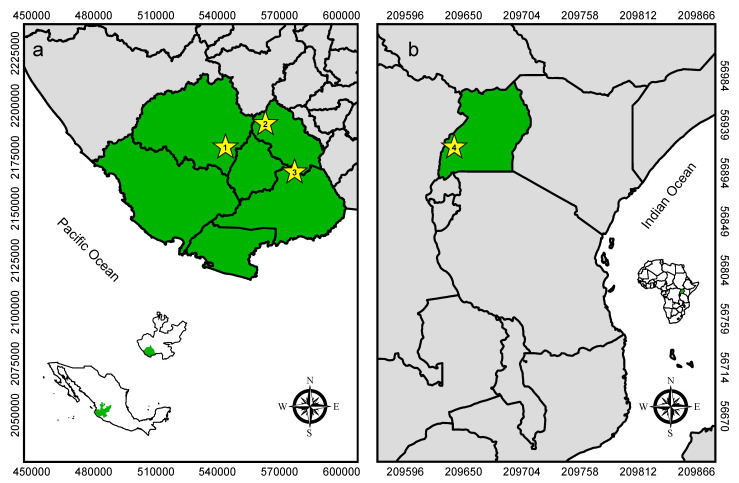
Maps of study sites. (**a**) Sampling locations in Jalisco, México (green area, shown with insets of México and Jalisco). Sites in México are 1: Villa Purificación, 2: Jalocote, and 3: Las Joyas Scientific Research Station. (**b**) Sampling location in Uganda (green, with inset of Uganda in Africa) in Kibale National Park (4), near the Ngogo Research Station. Numbers surrounding maps are latitudes and longitudes (UTM).

In México, we trapped bats in 3 locations in the southern part of Jalisco State: Villa Purificación (19°42′6.96″ N, 104°36′18.33″ E at 510 m elevation; semi-evergreen forest); Jalocote (19°48′55.95″ N, 104°24′37.05″ E at 1162 m elevation; dry deciduous forest with riparian vegetation); and Las Joyas Scientific Research Station (19°35′10.29″ N, 104°16′27.45″ E at 1950 m elevation; temperate high mountain forest) (Figure 1). Trapping occurred in 2018 during the months of January, February and April, respectively. We set mist nets (Avinet, Portland, ME, USA) at each location and opened them for four hours beginning at sunset. We gently removed netted bats and placed them into in cloth bags (one bat per bag) for processing. We collected bat flies using entomological tweezers and preserved them in 0.5 mL RNA*later* nucleic acid stabilization buffer (Thermo Fisher Scientific, Waltham, MA, USA) inside 1.2 mL cryovials (one vial per bat). After processing, we released bats on site.

In Uganda, we trapped bats in Kamwenge District at Ngogo research station within Kibale National Park (0°29’7.97″ N, 30°23’21.91″ E at 1550 m elevation; semideciduous montane forest) in July 2017, as previously described [14,17,18,19] (Figure 1). Briefly, we set mist nets at a large hollow tree roost and captured bats as they exited at dusk. We handled bats and collected bat flies as described above, and we released bats on site after processing.

### 2.2. Imaging of Bat Flies

We captured stacked images of bat flies using a KY-F75U digital camera (JVC, Yokohama, Japan) attached to a Z16 APO dissecting microscope (Leica Microsystems, Wetzlar, Germany) affixed with gooseneck illuminators (Ace 1, Schott Corporation, San Diego, CA, and Intralux 5000–1, Volpi Group, Schlieren, Switzerland). Images consisted of 15 stacked layers assembled using Auto Montage Pro software v5.01.005 (Synoptics LTD, Cambridge, UK).

### 2.3. DNA Barcoding of Bat Flies

We PCR-amplified and sequenced a 636-bp portion of the mitochondrial cytochrome oxidase subunit 1 (cox1) gene. We first surface-sterilized bat flies using dilute bleach [20] and homogenized them in 0.5 mL Hanks’ balanced salt solution in PowerBead tubes containing 2.38 mm metal beads (Qiagen, Hilden, Germany). We then centrifuged 50 µL of the resulting homogenates at 10,000× *g* for 10 min and extracted DNA from pelleted material using the DNeasy blood and tissue kit (Qiagen, Hilden, Germany). We PCR-amplified cox1 using barcoding primers LCO1490 (5′-GGTCAACAAATCATAAAGATATTGG-3′) and HC02198 (5′-TAAACTTCAGGGTGACCAAAAAATCA-3′) [21] and the HotStarTaq PCR system (Qiagen, Hilden, Germany), with the following cycling parameters: 95 °C for 15 min; 35 cycles of 94 °C for 30 s, 50 °C for 30 s, 72 °C for 1 min; 72 °C for 10 min, and an indefinite soak at 4 °C. We electrophoresed PCR products on 1% agarose gels stained with ethidium bromide, excised amplicons, gel-purified them using the Zymoclean Gel DNA Recovery Kit (Zymo Research, Irvine, CA, USA), and submitted purified amplicons for Sanger sequencing in both directions on 3730xl Genetic Analyzers (ThermoFisher, Waltham, MA, USA) at the University of Wisconsin-Madison Biotechnology Center. We proofread and assembled chromatograms using Sequencher 4.10.1 (Gene Codes, Ann Arbor, MI, USA) and queried the resulting sequences against DNA sequences of streblid bat flies available in GenBank database using the blastx homology searching algorithm [22].

### 2.4. Metagenomics and Phylogenetics

We processed bat flies for virus characterization using previously described methods [14,17]. Briefly, we extracted total nucleic acids from bat fly homogenates prepared as described above using the QIAamp Viral RNA Mini Kit (Qiagen, Hilden, Germany). We used the Superscript IV system (Thermo Fisher, Waltham, MA, USA) with random hexamers to reverse transcribe RNA to cDNA, and we prepared DNA libraries using the Nextera XT DNA sample preparation kit (Illumina, San Diego, CA, USA). We sequenced libraries on a MiSeq instrument (V2 chemistry, 300 cycle kit; Illumina, San Diego, CA, USA).

We quality-trimmed reads to ≥Q30 and length ≥50 and then assembled them de novo into contiguous sequences (contigs) using CLC Genomics Workbench version 20.1 (CLC Bio, Aarhus, Denmark). To characterize viruses, we queried raw reads and assembled contigs at the nucleotide and deduced amino acid sequence levels against sequences of viruses in GenBank using blastn and blastx, respectively [22], employing methods previously described for identifying viruses in bat flies [14,17].

To infer the phylogenetic positions of identified viruses, we aligned polymerase nucleotide sequences with related sequences in GenBank using a codon-based version of the Prank algorithm [23] with Gblocks [24] applied to remove poorly aligned regions, implemented in TranslatorX [25]. We then inferred phylogenies using PhyML 3.056 [26] with smart model selection [27] and 1000 bootstrap replicates to assess statistical confidence in clades. We displayed resulting phylogenetic trees using FigTree v1.4.4.

We measured viral concentrations as viral reads per million total reads per kilobase of target sequence (vRPM/kb), which is a metagenomic proxy for viral load that has been validated by comparison to quantitative real time polymerase chain reaction [28,29,30]. We calculated this measure for each virus in each sample by mapping reads to the polymerase gene sequence (or obtained portion thereof, when available) of each identified virus at 90% sequence identity then log_10_ transforming the results for ease of visualization.

## 3. Results

### 3.1. Bat Fly Identification

**Figure 2:**
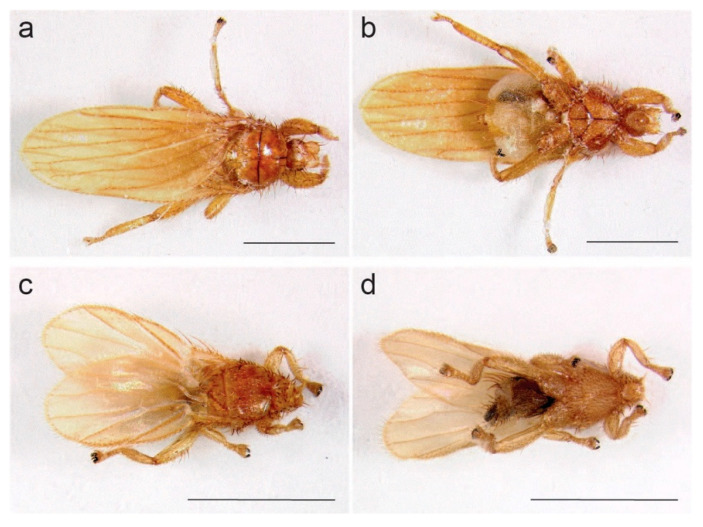
Images of streblid bat flies from México and Uganda. (**a**,**b**) The Méxican specimen (**a**: dorsal, **b**: ventral; 2.5× magnification and a patch size of 16) was collected from a Parnell’s mustached bat (*Pternotus parnellii* Gray, 1843) at Estación Científica Las Joyas, Sierra de Manantlán, Jalisco, on 17 January 2018. (**c**,**d**) the Ugandan specimen (**c**: dorsal, **d**: ventral; 4.0× magnification and a patch size of 18) was collected from a Noack’s roundleaf bat (*Hipposideros ruber* Noack, 1893) at Ngogo, Kamwenge District, on 1 July 2017. Scale bars = 1 mm.

We processed 12 bat fly samples (pools of between 1 and 6 flies per bat) from 9 species of bats in México and 12 bat fly samples from 1 species of bat in Uganda (Table S1). All bat flies analyzed had the characteristic morphology of the Streblidae (Figure 2). Sequences of the cox1 gene derived from the 24 samples matched five streblid genera, including *Aspidoptera*, *Megistopoda*, *Trichobioides,* and *Trichobius* in México, and *Nycterophilia* in Uganda (Table S1). In total, six samples from México were >99% identical to published strebid sequences, enabling species-level taxonomic assignment (*Aspidoptera phyllostomatis* Perty 1833, *Megistopoda aranea* Coquillett, 1899, and *Trichobius yunkeri* Wenzel, 1966; Table S1). Other sequences from México and all sequences from Uganda were between 97.64% and 80.82%, identical to published strebid sequences, such that we could not make definitive taxonomic assignments (Table S1). Furthermore, these more divergent sequences were, in many cases, phylogenetically intermediate between published sequences of known streblid species (data not shown), suggesting that these specimens represent un-sequenced or novel species.

### 3.2. Virus Characterization

**Figure 3:**
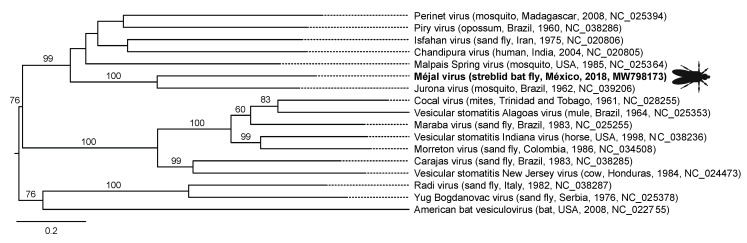
Maximum likelihood phylogenetic tree of vesiculoviruses based on L (polymerase) gene sequences. Virus names are followed (in parentheses) by host, country, year of collection, and GenBank accession number. Méjal virus, identified in the present study, is shown in bold type with silhouette of streblid bat fly. Numbers beside branches represent statistical confidence in clades based on 1000 bootstrap replicates; only bootstrap values ≥50% are shown. Scale bar = nucleotide substitutions per site.

Average sequencing depth was 1,018,337 (standard deviation 306,682) reads per bat fly sample after quality trimming, with average length of 126.3 base pairs (standard deviation 9.9). From these data, we identified four viruses in bat flies from México (Table 1). Méjal virus (*Rhabdoviridae*: *Vesiculovirus*) is most closely related to Jurona virus, isolated from mosquitoes in Brazil and of unknown medical significance [31] (Figure 3). The other viruses in this clade are vectored by mosquitoes and sand flies, and Chandipura virus, Piry virus, and Ishafan virus can infect humans, the former two causing febrile illness [32,33,34]. This clade, in turn, is most closely related to the clade of vesiculoviruses containing the vesicular stomatitis viruses (VSV; Figure 3), which are important livestock pathogens that occasionally infect people [35,36].

**Figure 4:**
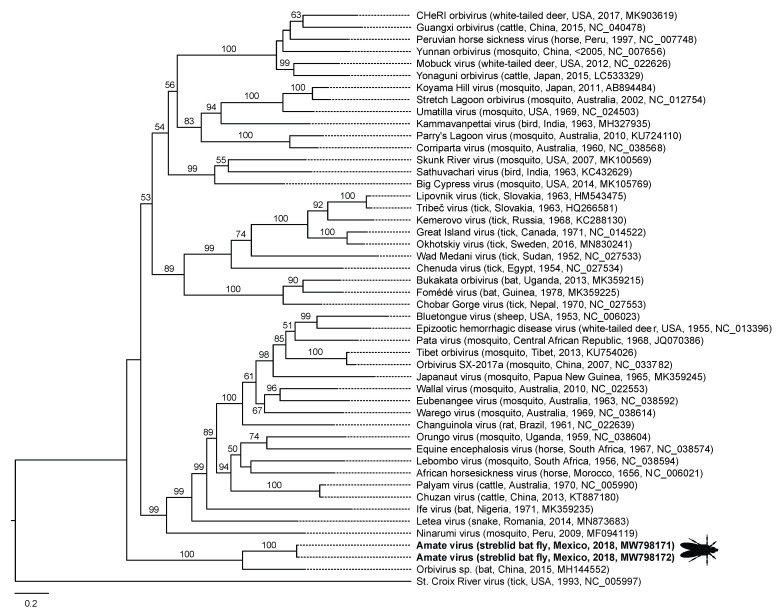
Maximum likelihood phylogenetic tree of orbiviruses based on VP1 (polymerase) gene sequences. Virus names are followed (in parentheses) by host, country, year of collection, and GenBank accession number. The two closely related variants of Amate virus (98.7% nucleotide identity in the VP1 gene) identified in the present study, are shown in bold type with silhouette of streblid bat fly. Numbers beside branches represent statistical confidence in clades based on 1000 bootstrap replicates; only bootstrap values ≥50% are shown. Scale bar = nucleotide substitutions per site.

Additionally, in México we found two closely related variants of Amate virus (*Reoviridae*: *Orbivirus*). The genus *Orbivirus* contains several important ungulate pathogens, most notably the globally emerging and economically significant agricultural pathogen bluetongue virus [37,38] (Figure 4). Amate virus is most closely related (based on segment 1) to an unnamed virus from a black-bearded tomb bat (*Taphozous melanopogon* Temminck, 1841) in China (GenBank MH144552), together forming a clade that is most closely related to the mosquito- and tick-borne orbiviruses (Figure 3), some of which are zoonotic [39,40]. The other two viruses from Mexican bat flies (jalimé iflavirus 1 and jalimé partiti-like virus 1) are members of viral groups known only from broad metagenomic surveys of invertebrates (Table 1; [41]).

**Figure 5:**
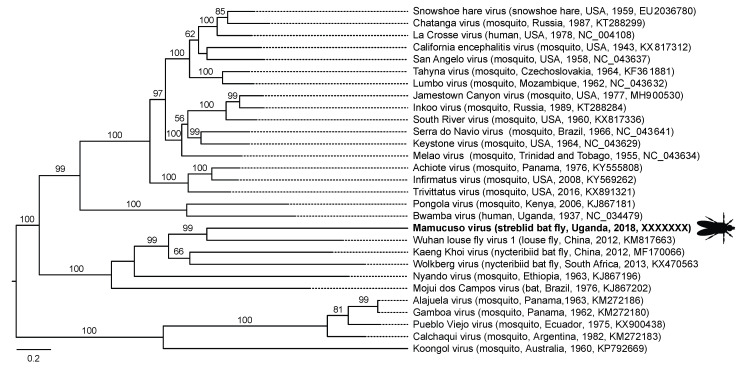
Maximum likelihood phylogenetic tree of orthobunyaviruses based on L (polymerase) gene sequences. Virus names are followed (in parentheses) by host, country, year of collection, and GenBank accession number. Mamucuso virus, identified in the present study, is shown in bold type with silhouette of streblid bat fly. Numbers beside branches represent statistical confidence in clades, based on 1000 bootstrap replicates; only bootstrap values ≥50% are shown. Scale bar = nucleotide substitutions per site.

We identified three viruses in bat flies from Uganda (Table 1). Mamucuso virus (*Peribunyaviridae*: *Orthobunyavirus*) is a member of a genus containing vector-borne pathogens known to cause severe encephalitis in humans and other mammals (Figure 3) [42,43,44]. Within the orthobunyaviruses, mamucuso virus is part of a subclade of viruses from nycteribiid bat flies and louse flies (based on the L segment), also containing the mosquito-vectored human pathogen Nyando virus [42] (Figure 5). This clade is most closely related to the clade containing Bwamba virus, a highly prevalent cause of human febrile illness across Africa [42,45], and the less common Pongola virus, which also causes human febrile illness [42,46]. The clade containing mamucuso virus is equally closely related to the globally expanding and neuropathogenic California serogroup of orthobunyaviruses [42]. Another virus from Ugandan bat flies, kamu rhabdovirus 1, appears to be a divergent member of the family *Rhabdoviridae*, although only a small fragment (609 bases) of the nucleocapsid gene could be obtained (Table 1). The other virus from Ugandan bat flies, kamu toti-like virus 1, is a member of a viral group known only from broad metagenomic surveys of invertebrates (Table 1; [41]).

### 3.3. Virus Prevalence and Load

**Figure 6:**
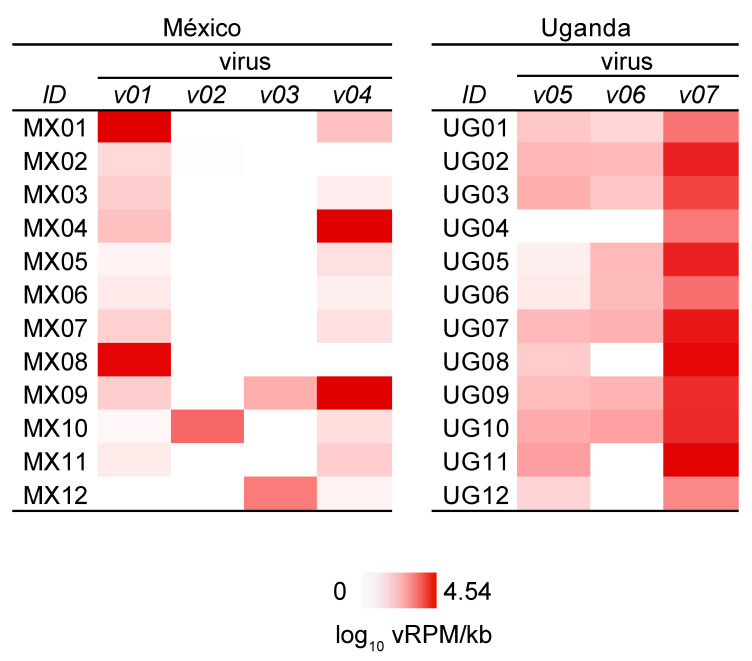
Heatmap of viral loads in streblid bat flies from México and Uganda. Data are log_10_ viral reads per 10^6^ total reads per kilobase of target sequence for each virus in each bat fly (MX01-MX12 and UG01-UG12): méjal virus (v01), Amate virus (v02), jalimé iflavirus 1 (v03), jalimé partiti-like virus 1 (v04), mamucuso virus (v05), kamu rhabdovirus 1 (v06), and kamu toti-like virus 1 (v07).

All bat fly samples analyzed harbored at least one virus, with an average of 2.1 viruses per sample in México, 2.7 viruses per sample in Uganda, and 2.4 viruses per sample overall. Prevalence of the 7 viruses identified in México and Uganda varied widely (Figure 6), ranging from 2/12 (16.7%) for méjal virus and jalimé iflavirus 1 to 12/12 (100%) for kamu toti-like virus 1. Prevalence of 11/12 (91.7%) for Amate virus and mamucuso virus, 10/12 (83.3%) for jalimé partiti-like virus 1, and 8/12 (66.7%) for kamu rhabdovirus 1 were intermediate. Similarly, viral loads varied over four orders of magnitude among viruses and samples, from 0 to 4.54 log_10_ vRPM/kb (Figure 4). Kamu toti-like virus 1 had the highest average viral load across positive samples (3.42 log_10_ vRPM/kb) followed by jalimé iflavirus 1 (1.93), jalimé partiti-like virus 1 (1.47), méjal virus (1.39), amate virus (1.32), kamu rhabdovirus 1 (1.12), and mamucuso virus (1.10).

## 4. Discussion

Our results show that New World and Old World streblid bat flies host viruses related to medically and agriculturally important pathogens. In streblids from México, we found méjal virus, a rhabodovirus in the genus *Vesiculovirus*. The closest known relative of méjal virus is Jurona virus, isolated in 1962 from mosquitoes in Brazil but with no clear association with disease [47]. However, vesiculoviruses in the clade to which méjal virus belongs (Figure 3) infect humans and can cause disease, as has been shown for Chandipura virus, an emerging pathogen that causes encephalitis in people (mainly children) in India [34], Piry virus, which has caused serious febrile illness in humans infected through laboratory accidents [33], and Ishafan virus, which seroprevalence studies show has infected up to 86% of people in communities in Iran [32]. Other vesiculoviruses are important to animal health and agriculture. Notably, the vesicular stomatitis viruses (VSV) are endemic zoonoses in México, South and Central America and occasionally infect livestock in the United States to cause epizootics of vesicular stomatitis, an economically important disease resembling foot and mouth disease [35].

In streblids from México, we also found Amate virus, a reovirus in the genus *Orbivirus*. Orbiviruses are genetically and antigenically diverse human and animal arboviruses that are emerging globally [39,40]. Notably, bluetongue virus is a major emerging pathogen of wild and domestic ungulates worldwide that can also infect dogs and is transmitted either by hematophagous midges of the genus *Culicoides* or, in the case of certain emerging variants, horizontally [37]. Amate virus and its sister taxon, an incompletely described orbivirus from a black-bearded tomb bat (*T. melanopogon*) in China, are notably divergent from the tick-borne and mosquito borne orbiviruses (Figure 4), suggesting a lineage of bat-infecting orbiviruses with bat flies as vectors. Other bat-associated orbiviruses included in Figure 3 include Japanaut virus from a southern blossom bat (*Syconycteris crassa* Thomas, 1895) and culicine mosquitoes in New Guinea [48], Ife virus from a straw-colored fruit bat (*Eidolon helvum* Kerr, 1792) in Nigeria, Cameroon, and the Central African Republic [49], and the sister taxa Fomédé virus and Bukakata orbivirus from a dwarf slit-faced bat (*Nycteris nana* Andersen, 1912) in Guinea [50], and an Egyptian fruit bat (*Rousettus aegyptiacus leachii* Smith, 1892) in Uganda [51], respectively. Heramatsu orbivirus was also isolated from an eastern long-fingered bat (*Myotis macrodactylus* Temminck, 1840) in Japan [52] but remains incompletely sequenced. Interestingly, Bukakata virus and Fomédé virus cluster within the tick-born orbivirus clade (Figure 3), and serum neutralization assays of 54 bat samples from Bolivia identified one *M. nigricans* Schinz, 1821, and one *Noctilio labialis* Hershkovitz, 1949, to have been exposed to Matucaré virus, another incompletely sequenced tick-borne orbivirus [53]. Bat ticks, like bat flies, are specialized for life on bats [54,55], such that orbivirus transmission through bat tick vectors could explain these observations.

In streblid bat flies from Uganda, we found mamucuso virus, a peribunyavirus in the genus *Orthobunyavirus*. Orthobunyaviruses are a large group of arboviruses with a worldwide and expanding distribution that cause severe central nervous system disease in vertebrates [42,43,44]. Mamucuso virus is part of a group of viruses associated with bats and their vectors (Figure 5). The closest relative of mamucuso virus is Wuhan louse fly virus 1, found in a louse fly (Hippoboscoidea: *Hippoboscidae*), which are obligate hematophagous ectoparasites closely related to bat flies [56]. Mamucuso virus’s next nearest relatives are Kaeng Khoi virus and Wolkberg virus (Figure 5), both of which infect nycteribiid bat flies [10,11,12], with Kaeng Khoi virus being apparently pathogenic in bats [57]. Interestingly, Kaeng Khoi virus has also been isolated from bat bugs (*Stricticimex parvus* Ueshima) in Thailand, suggesting that it has a broad vector range among hematophagous arthropods [58]. Nyando virus, the only mosquito-vectored virus in the mamucuso virus clade and the sole member of its serogroup, causes febrile illness and is highly prevalent (based on serology) in equatorial African countries, including Uganda [59]. Mojui de Campos virus, isolated from an adult bat (species not specified) in Brazil in 1975, was not found in 31,645 mosquitoes of six genera collected from the same location at the same time [60]. Thus, our results and those of others support the existence of a group of *Orthobunyavirus* that are transmitted by hippoboscoid flies rather than by mosquitoes [59].

In addition to the vesiculoviruses, orbiviruses, and orthobunyaviruses, we found four RNA viruses from unclassified genera (Table 1) that are most likely vertically transmitted insect viruses characteristic of the highly diverse invertebrate “virosphere” [41,61]. A possible exception is kamu rhabdovirus 1, a divergent rhabdovirus that we could not place phylogenetically due to limited sequence data but that may ultimately cluster with other medically important rhabdoviruses. Like many invertebrates, therefore, streblid bat flies host viruses from medically important lineages and other viral taxa specific to invertebrates [62].

In México, we sampled 3 sites and 9 species of bats, parasitized by 4 genera of bat flies in which we found 4 viruses (Table 1 and Table S1) and an average of 2.1 viruses per sample. In Uganda, we sampled 1 site and 1 species of bat parasitized by a single genus (and putative species) of bat fly in which we nevertheless found 3 viruses (Table 1 and Table S1) and an average of 2.7 viruses per sample. Prevalence and loads varied markedly but were comparable among viruses in both countries. Thus, despite greater geographic range, bat diversity, and bat fly diversity sampled in México than in Uganda, the richness and diversity of viruses identified was similar in both countries. These results support our previous findings that nycteribiid bat flies from a few or even a single location can host a greater diversity of viruses than would be predicted from the bats they parasitize [14]. The imperfect specificity of viruses for bat flies and of bat flies for bats in México was surprising (Figure 6; Table S1). Our study sites in México and Uganda are both biodiversity “hot spots” where host switching by bat flies may occur due, for example, to multispecies bat roosting [14,63,64], perhaps explaining these observations.

In the case of the orbiviruses and orthobunyaviruses, phylogenetic trees imply the existence of heretofore unrecognized transmission cycles involving bats and bat flies. The tightly co-evolved relationships between bat flies and their bat hosts may predispose the evolution of “hyperparasitism,” or “parasitism of parasites”, in which symbionts such as the viruses documented herein become adapted to the codependent life history of host and parasite [65]. The evolution of hyperparasitism has been documented for nycteribiid bat flies and Laboulbeniales fungi [66] and for nycteribiid bat flies and rhabdoviruses of the genus *Ledantevirus* [14]. Our results suggest that communities of hyperparasitic viruses have also co-evolved with streblid bat flies and exist on at least two continents. If so, bat flies may be “missing ecological links” between bats and other mammals with respect to viral transmission [14,65]. Many viruses are called “bat associated” because they are ecologically associated with bats but have rarely or never been found in bats themselves. The viruses we have documented in streblid bat flies support the notion that some “bat associated” viruses may actually be viruses of bat ectoparasites.

Bat flies take blood meals from bats nearly constantly [1,5,67], such that transmission of bat fly viruses to bats might occur even if transmission is inefficient. Bat flies also bite humans opportunistically (as do other Hippoboscoidea) [1,56,67], suggesting a mechanism for zoonotic transmission. Several zoonotic rhabdoviruses, for example, have rarely or never been found in bats but they or their relatives have been found bat flies [14,17]. Viruses of bat flies may, therefore, not fall neatly into categories, such as “vector-borne” or “vertically transmitted”. Rather, they may be adapted to modes of transmission along a spectrum from vertical (fly to fly) to vector-borne (fly to mammal to fly) to directly transmitted (bat to bat, or bat to other mammal). An example of a virus with a similarly heterogeneous mode of transmission is La Crosse virus, a relative of mamucuso virus within the California serogroup of orthobunyaviruses (Figure 5), which is maintained in nature through a combination of vector-borne and transovarial transmission [68]. Determining whether the viruses discovered here infect bats, bat flies, or both, and with what efficiency, would necessitate additional study and would likely require experimental infections.

Our results also suggest an explanation for the enigmatic epidemiology of vesicular stomatitis. Despite the economic costs of vesicular stomatitis, the ecological mechanism by which the virus moves from Central or South America to the western USA remains unknown [35,69]. In Colombia, Alagoas serotype vesicular stomatitis virus has been isolated from naturally infected phlebotomine sand flies (*Lutzomyia* spp.) and can be transmitted through biting and transovarially under experimental conditions [70]. In the eastern USA (Ossabaw Island, Georgia), VSV New Jersey has been found at relatively high titer in *L. shannoni* Dyar [71,72], and *L. shannoni* can transmit the virus to rodents through biting and to a small percentage of progeny via transovarial transmission under experimental conditions [73]. However, in the western USA (New Mexico and Colorado), sandflies have been found only in small numbers on or near livestock ranches with histories of vesicular stomatitis [74], and no relationship between sand flies and VSV has been established in this region. Furthermore, there is no evidence of which we are aware that VSV is repeatedly transported in suspect vectors moving unaided from endemic areas in Mexico, Central or South America to the western USA.

We speculate that vesiculoviruses such as VSV might enter the USA on migratory bats and their ectoparasites and then become locally established in alternative vectors such as sand flies. Bat migration is an underappreciated mechanism for the long-distance transport of bat-associated pathogens [75]. Such a scenario could explain why VSV outbreaks in the southwestern United States occur in summer [69], coincident with the northward migration of certain bats (e.g., the Mexican free-tailed bat, *Tadarida brasiliensis mexicana* Saussure, 1860) into the USA to establish breeding colonies [76], as well as how the virus might initially be transmitted to livestock (e.g., via bat flies feeding on alternative hosts). In support of this idea, Malpais Spring virus, within the méjal virus clade (Figure 3), is another vector-borne virus that infects wild and feral ungulates in New Mexico, USA, where VSV outbreaks also occur [31,77].

Our study has certain inherent limitations. First, we could only examine a small number of bat flies from México and Uganda. The global diversity of streblid bat fly viruses is, therefore, likely much higher than we have documented here. Second, identifying viruses using metagenomics does indicate whether those viruses actively replicate in bat flies. Certain bat flies had very high viral loads with sequences yielding complete viral genomes with deep coverage, suggesting replication (Figure 6), but experimental infection studies would be necessary to demonstrate replication definitively. Third, it is unclear whether the viruses we describe in streblid bat flies infect bats, either through classical vector-borne transmission or through alternative, heterogeneous transmission cycles, as described above. Unfortunately, matched samples from parasitized bats were not available for analysis.

Despite these caveats, our data strongly suggest that streblid bat flies are underappreciated reservoirs of viral diversity. Bat flies occupy a unique niche that predisposes them towards infection with, and transmission of, viruses with ecological and evolutionary links to bats. In this light, we note that bats host other types of highly co-evolved ectoparasites, such as bat fleas, bat mites, bat ticks, and even bat leeches [54,55,78]. These parasites may also host hyperparasitic viruses of relevance to human and animal health. Investigating the full taxonomic range of bat ectoparasites may therefore shed new light on the ecology, evolution and epidemiology of enigmatic “bat associated” viruses.

## Figures and Tables

**Table 1:** Viruses identified in streblid bat flies from México and Uganda.

Country	Virus ^1^	Accession	Genome	Closest Match ^2^	Family ^3^	Genus ^3^	% Identity ^4^
México	Amate virus	MW798171MW798172	dsRNA, segmented	Orbivirus sp (*Taphozous melanopogon* bat, China, 2018, MH144552)	*Reoviridae*	*Orbivirus*	68.65 (78, 0.0)
México	Méjal virus	MW798173	−ssRNA, linear	Jurona virus (*Haemagogus* sp. mosquito, Brazil, 1962, NC_039206)	*Rhabdoviridae*	*Vesiculovirus*	68.60 (91, 0.0)
México	Jalimé iflavirus 1	MW798174	+ssRNA, linear	Varroa destructor virus 2 (*Varroa destructor* mite, Israel, 2014, NC_040601)	*Iflaviridae*	unclassified	72.04 (42, 3e-53)
México	Jalimé partiti-like virus 1	MW798175	dsRNA	Wuhan insect virus 24 (unspecified insects, China, 2013, KX884086)	unclassified	unclassified	68.42 (85, 7e-109)
Uganda	Mamucuso virus	MW798176	−ssRNA, segmented	Wuhan Louse Fly Virus 1 (unidentified Hippoboscoidea, China, 2012, KM817663)	*Peribunyaviridae*	*Orthobunyavirus*	69.45 (96, 0.0)
Uganda	Kamu rhabdovirus 1	MW798177	−ssRNA, linear	American bat vesiculovirus (*Eptesicus fuscus* bat, USA, 2008, JX569193)	*Rhabdoviridae*	unclassified	72.90 (22, 9e-13)
Uganda	Kamu toti-like virus 1	MW798178	dsRNA	Hubei toti-like virus 22 (unspecified insects, China, 2013, KX882954)	unclassified	unclassified	76.79 (5, 2e-9)

^1^ Etymology of virus names is as follows. “Amate” refers to a culturally important tree (*Ficus obtusifolia*) native to México in which many of the sampled bats roost. “Méjal” is a hybridization of “México” and “Jalisco.” “Jalimé” is a hybridization of “Jalisco” and “México.” “Mamucuso” refers to a culturally important tree (*Ficus mucuso*) native to Uganda, with “ma” referring to the local Tooro word for “mother” (the bats were sampled from a large, hollow specimen of this tree species known locally as the “mother mucuso”). “Kamu” is a hybridization of “Kamwenge” and “Uganda.”. ^2^ Closest match in the GenBank nucleotide database (source, location, date and accession number of closest match in parentheses). ^3^ Family and genus of the closest match in the GenBank database. ^4^ Percent nucleotide sequence identity to the closest match in the GenBank database (query cover and E-value in parentheses).

## Data Availability

All data supporting reported results can be found herein. DNA sequence data are available in GenBank (MW792194-MW792215 and MW798171-MW798178).

## Supplementary Materials

The following are available online at https://www.mdpi.com/article/10.3390/v13050860/s1, Table S1: Classification of streblid bat flies based on mitochondrial cytochrome oxidase subunit I DNA sequences.

## Animal Use Statement

The study was approved by the Institutional Animal Care and Use Committees of Universidad de Guadalajara and the University of Wisconsin-Madison (Protocol V005734-R01).
