# Supplementary material for: Bat Flies of the Family Streblidae (Diptera: Hippoboscoidea) Host Relatives of Medically and Agriculturally Important “Bat-Associated” Viruses
Source: Viruses. 2021 May 8;13(5):860. doi: 10.3390/v13050860 (PMC8150819; doi:10.3390/v13050860)
Supplement: Supplementary file 1 [file viruses-13-00860-s001.zip › viruses-1176929-supplementary.pdf]

Table S1. Classification of streblid bat flies based on mitochondrial cytochrome oxidase subunit I DNA sequences

ID	Country	Site	Host bat species	Accession	Closest match (accession) [1]	% identity [2]
JAL01	México	Las Joyas	*Pteronotus parnellii*	MW792194	*Trichobius sparsus* (MH282325.1)	97.64
JAL02	México	Las Joyas	*Myotis thysanodes*	nd [3]	nd [3]	nd [3]
JAL03	México	Jalocote	*Artibeus jamaicensis*	MW792195	*Megistopoda aranea* (MH282060.1)	99.21
JAL04	México	Jalocote	*Artibeus jamaicensis*	MW792196	*Megistopoda aranea* (MH282060.1)	99.21
JAL05	México	Jalocote	*Artibeus lituratus*	MW792197	*Megistopoda aranea* (MH282060.1)	99.21
JAL06	México	Jalocote	*Artibeus jamaicensis*	MW792198	*Aspidoptera phyllostomatis* (MH282004.1)	99.69
JAL07	México	Villa Purificación	*Pternotus parnellii*	MW792199	*Trichobius yunkeri* (MH282371.1)	99.84
JAL08	México	Villa Purificación	*Pternotus parnellii*	MW792200	*Trichobius yunkeri* (MH282369.1)	100
JAL09	México	Villa Purificación	*Sturnira lilium*	MW792201	*Aspidoptera phyllostomatis* (MH282004.1)	97.48
JAL10	México	Villa Purificación	*Glossophaga commissarisi*	MW792202	*Trichobius uniformis* (MH282328.1)	97.64
JAL11	México	Villa Purificación	*Dermanura watsoni*	MW792203	*Trichobioides perspicillatus* (MH282206.1)	80.82
JAL12	México	Las Joyas	*Sturnia ludovici*	nd [3]	nd [3]	nd [3]
NGO01	Uganda	Ngogo	*Hipposideros ruber*	MW792204	*Nycterophilia parnelli* (KY882260.1)	86.99
NGO02	Uganda	Ngogo	*Hipposideros ruber*	MW792205	*Nycterophilia parnelli* (MH282091.1)	86.30
NGO03	Uganda	Ngogo	*Hipposideros ruber*	MW792206	*Nycterophilia parnelli* (MH282091.1)	86.30
NGO04	Uganda	Ngogo	*Hipposideros ruber*	MW792207	*Nycterophilia parnelli* (MH282091.1)	86.93
NGO05	Uganda	Ngogo	*Hipposideros ruber*	MW792208	*Nycterophilia parnelli* (MH282091.1)	86.30
NGO06	Uganda	Ngogo	*Hipposideros ruber*	MW792209	*Nycterophilia parnelli* (MH282091.1)	86.30
NGO07	Uganda	Ngogo	*Hipposideros ruber*	MW792210	*Nycterophilia parnelli* (MH282091.1)	85.85
NGO08	Uganda	Ngogo	*Hipposideros ruber*	MW792211	*Nycterophilia parnelli* (KY882260.1)	86.99
NGO09	Uganda	Ngogo	*Hipposideros ruber*	MW792212	*Nycterophilia parnelli* (MH282091.1)	85.98
NGO10	Uganda	Ngogo	*Hipposideros ruber*	MW792213	*Nycterophilia parnelli* (MH282091.1)	86.30
NGO11	Uganda	Ngogo	*Hipposideros ruber*	MW792214	*Nycterophilia parnelli* (MH282091.1)	86.30
NGO12	Uganda	Ngogo	*Hipposideros ruber*	MW792215	*Nycterophilia parnelli* (MH282091.1)	86.30

[1] Closest match to members of the family *Streblidae* in the GenBank nucleotide database identified using the blastn homology searching algorithm

[2] Percent nucleotide identity to the closest match in GenBank

[3] nd = not determined
